# Creatinine Index as a Surrogate of Lean Body Mass Derived from Urea Kt/V, Pre-Dialysis Serum Levels and Anthropometric Characteristics of Haemodialysis Patients

**DOI:** 10.1371/journal.pone.0093286

**Published:** 2014-03-26

**Authors:** Bernard Canaud, Alexandre Granger Vallée, Nicolas Molinari, Leila Chenine, Hélène Leray-Moragues, Annie Rodriguez, Lotfi Chalabi, Marion Morena, Jean-Paul Cristol

**Affiliations:** 1 Nephrology, Dialysis and Intensive Care Unit, CHRU, Montpellier, France; 2 Dialysis Research and Training Institute, Montpellier, France; 3 UMR 729 MISTEA, DIM, CHRU, Montpellier, France; 4 AIDER, Montpellier, France; 5 Biochemistry Laboratory, CHRU, Montpellier, France; 6 UMR 204, University of Montpellier I, Montpellier, France; University Medical Center Groningen and University of Groningen, Netherlands

## Abstract

**Background and Objectives:**

Protein-energy wasting is common in long-term haemodialysis (HD) patients with chronic kidney disease and is associated with increased morbidity and mortality. The creatinine index (CI) is a simple and useful nutritional parameter reflecting the dietary skeletal muscle protein intake and skeletal muscle mass of the patient. Because of the complexity of creatinine kinetic modeling (CKM) to derive CI, we developed a more simplified formula to estimate CI in HD patients.

**Design, Setting, Participants & Measurements:**

A large database of 549 HD patients followed over more than 20 years including monthly CKM-derived CI values was used to develop a simple equation based on patient demographics, predialysis serum creatinine values and dialysis dose (_sp_Kt/V) using mixed regression models.

**Results:**

The equation to estimate CI was developed based on age, gender, pre-dialysis serum creatinine concentrations and _sp_Kt/V urea. The equation-derived CI correlated strongly with the measured CI using CKM (correlation coefficient  = 0.79, p-value <0.001). The mean error of CI prediction using the equation was 13.47%. Preliminary examples of few typical HD patients have been used to illustrate the clinical relevance and potential usefulness of CI.

**Conclusions:**

The elementary equation used to derive CI using demographic parameters, pre-dialysis serum creatinine concentrations and dialysis dose is a simple and accurate surrogate measure for muscle mass estimation. However, the predictive value of the simplified CI assessment method on mortality deserves further evaluation in large cohorts of HD patients.

## Introduction

Protein-energy wasting (PEW) is highly prevalent among the hemodialysis (HD) population [1], [2]. Several studies have underlined the prognostic significance of PEW as a strong predictor of morbidity and mortality independently of dialysis adequacy in HD patients [3]–[5]. Protein nutritional status is determined by markers of visceral and somatic protein stores [6]. Although serum albumin concentration, a visceral protein indicator, is routinely assessed and is inversely associated with mortality in HD patients [3], [4], [7], [8], it is an insensitive confounding factor being an indicator of both inflammation and nutritional status [9], [10]. In addition to assessment of visceral proteins, the evaluation of somatic protein status by determination of muscle mass is crucial and commonly used for nutritional assessment of dialysis patients [6]. Reduced muscle mass and lean body mass (LBM) significantly correlate with higher mortality in HD patients [11]. The association of serum creatinine level with LBM and an inverse correlation between serum creatinine concentration and mortality in HD patients [4], [8], [12] support the use of serum creatinine as a nutritional and muscle mass marker and a predictor of clinical outcomes in these patients [13]. Nevertheless, serum creatinine concentration could be influenced by patient muscle mass, dietary protein intake, hydration status, dialysis clearance and the presence of residual renal function [14], [15].

An accurate measure of LBM by using computed tomographic (CT) or magnetic resonance imaging (MRI) of muscle mass, total body potassium counting and deuterium dilution techniques [16], [17] is cumbersome, expensive and not readily accessible in clinical practice. The estimation of LBM by creatinine kinetic modeling (CKM) has been validated as a convenient and reliable method of assessment of muscle mass and protein nutritional status in HD patients. CKM is based on the principle that creatinine generation is proportional to LBM in stable dialysis patients who have a constant protein/meat intake. Creatinine index (CI) is defined as normalized creatinine production rate, which is equal to the sum of creatinine excretion rate (dialytic removal and urinary excretion) and metabolic degradation rate in the steady state [18], [19]. Furthermore, CI derived from CKM as a marker of muscle mass has been recognized as a powerful prognostic indicator of long-term all-cause and cardiovascular mortalities in HD patients [20], [21]. The application of standard CKM requires post-dialysis serum creatinine concentration and dialysate collection to compute creatinine generation rates. Formulas for the prediction of post-dialysis serum creatinine levels and creatinine generation rates have been devised and validated in HD patients [20]. However, the complex mathematical formula requiring computer programs to precisely calculate CI poses a major obstacle to its application in routine clinical practice. Thus, in this study, we aimed to develop and validate a simple formula to substitute the complex equations or formal CKM to calculate CI using patient demographics and classical dialysis dose estimated by _sp_Kt/V urea in HD patients.

## Materials and Methods

### Ethics Statement

The manuscript describes an observational non-interventional study, so written informed consent was not required. According to the French Law, the study has been registered at “Ministère de l′Enseignement Supérieur et de la Recherche” after approval by our institution ethical committee (Comité de Protection des Personnes Sud Méditerranée IV, Montpellier, France) with the following number DC-2008-417. The study was conducted according to the principles of the Declaration of Helsinki and in compliance with International Conference on Harmonization/Good Clinical Practice regulations.

### Data sources

We used a large database (available since 1988) of prevalent and incident HD patients treated in two HD units in Montpellier, France (Lapeyronie University Hospital and the AIDER-Montpellier; Association pour l′Installation à Domicile des Épurations Rénales). Dialysis quantification including formal urea kinetic modeling (UKM) and CKM was routinely evaluated on a monthly basis on the mid-week HD sessions. HD charts and laboratory results were collected in an electronic primary care record only available to physicians and nurses from the dialysis centers. These primary data could not be publicly available. The data were collected from 549 patients undergoing HD for more than 3 months including 16,547 monthly assessments of formal UKM and CKM.

### Data collection, procedures and laboratory analysis

Medical records were reviewed for age, gender, ethnicity, pre- and post-dialysis weights, treatment modalities and time on dialysis. Formal UKM and CKM were routinely performed during the mid-week dialysis session of the first week of the month in all HD patients in our dialysis units. Pre- and post-dialysis blood samples were collected immediately before, at the end of dialysis sessions (2 minutes after stopping blood pump and before rinsing back of dialyzers) and at the beginning of the following dialysis session. During the first hour of dialysis session, blood samples were drawn from the arterial and venous blood lines and effluent dialysate sample was simultaneously taken. All blood and dialysate samples were measured for urea and creatinine concentrations by an enzymatic method using an automatic analyzer.

### Calculations

A single-pool variable-volume model was assumed to calculate creatinine distribution volume (Vc), creatinine appearance rate (Gc) and CI. In this model [18], CI, reflecting creatinine generation rate, is assumed to be equal to the sum of creatinine appearance rate (Gc) and metabolic degradation rate of creatinine (Dc). The dialysis adequacy (_sp_Kt/V urea) was determined by formal UKM applying a single-pool variable-volume model to pre-dialysis, post-dialysis and pre-dialysis of the next dialysis session serum urea concentrations to calculate urea distribution volume (Vu), urea appearance rate (Gu) using an in vivo hemodialyzer urea clearance (Kdu) and accounting for residual renal function (Kr) [18], [22]. Equilibrated Kt/V urea (eKt/V urea) estimated by using simplified equations, as proposed by Daugirdas et al. [23], and urea reduction ratio (URR) were also evaluated to determine dialysis dose. The database, which contained 16,547 values for CKM-derived CI from 549 HD patients, was used to develop and validate the equation. Patients with a significant diuresis (>500 ml urine/24 hours) and/or residual renal function (>2 ml/min) were excluded from the analysis to prevent complicating the final formula.

### Mathematical modeling to derive CI formulae from serum creatinine concentrations, _sp_Kt/V urea and anthropometric parameters

CKM-derived CI was measured at different time points among different HD patients. Mixed regression models were applied to generate formulae for CI prediction from dialysis dose and patient demographics. In this model, measured CI as an outcome variable Y was represented as a function of an intercept, whereas dialysis dose and demographic parameters were represented as predictor variables X. A random error (ε) was assumed in this model. Mixed regression model can be expressed as follows:
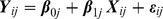
where *Y*_ij_ and *X*_ij_ are outcome variable and predictor variable at time i for subject j respectively and *β*_0j_ as well as *β*_1j_ are regression coefficients. Several predictor variables including patient demographics and dialysis dose were assumed according to an additive model. A regression model and CI estimations were obtained by statistical estimations of regression coefficients. Different formulae were developed and evaluated including those with dry weight and weight loss. We selected the simplest formula that provided the most accurate result. As results, the mixed regression model that best fits in our study is shown as follows:
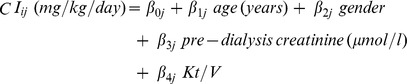



### Validation of modeling and error estimate in a large cohort of dialysis patients

We estimated the validity of our equation by examining differences between measured CI and CI predicted using our simplified equation based on 16,547 values for measured CI from 549 HD patients. Regression analysis and Bland-Altman analysis were performed to determine the differences between measured CI and predicted CI and estimate the mean prediction error.

### Statistical analyses

Multivariate mixed model analysis was performed using a generalized linear mixed-effect model for repeated measures. Variables were selected if p-value was less than 0.20 in the univariate analysis and a stepwise procedure was used to select potential variables for the final model. Regression and Bland-Altman analyses were performed to validate the equation. The continuous variables were expressed as mean ± standard deviation for normally distributed variables or median [interquartile range] for non normally distributed variables. *P*-values of <0.05 were considered to be statistically significants. Data were analyzed using R version 2.13.0.

## Results

General characteristics of the HD population used for equation generation are shown in **Table 1**. The study population included slightly higher proportion of men of Caucasian origin. The median age of the patients was 70.1 years old. Causes of CKD were glomerulonephritis (n = 92, 16.8%), diabetic and hypertensive nephropathy (n = 99; 18.1%), angiosclerosis and hypertensive nephropathy (n = 91; 16.6%), infectious/obstructive interstitial nephropathy (n = 72; 13.1%), renal neoplasia (n = 16; 2.9%), genetic/congenital cause (n = 61; 11.0%), unknown cause (n = 65; 11.8%), systemic disease (n = 49; 8.9%), other cause (n = 4; 0.8%). As previously mentioned, HD patients had no residual renal function at the time of measuring CI. The average creatinine indices (CIs) for men and women were 21.0 and 18.8 mg/kg/day respectively.

**Table 1:** Clinical characteristics of hemodialysis patients used for the development of a simplified equation for the calculation of creatinine index.

Parameters	
**Number**	549
**Men, n (%)**	330 (60.1)
**Age (years)**	70.1 [57.0–78.7]
**Diabetes, n (%)**	143 (26.0)
**Chronic obstructive pulmonary disease, n (%)**	79 (14.4)
**Chronic hepatitis, n (%)**	86 (15.7)
**History of kidney transplantation, n (%)**	89 (16.3)
**Ischemic cardiopathy, n (%)**	154 (28.1)
**Renal replacement modality (%HDF/HD)**	59.8/40.2
**Post-dialysis weight (kg)**	64.7 [55.4–75.2]
**Treatment time (min)**	219±33
**Pre-dialysis serum creatinine concentrations (μmol/l)**	733 [616–857]
**Residual renal function (ml/min)**	0
**Urea reduction ratio (%)**	78 [75–81]
**_sp_Kt/V urea**	1.8 [1.6–2.0]
**_eq_Kt/V urea**	1.6 [1.4–1.7]
**Creatinine index (mg/kg/day)**	18.9 [15.4–22.9]
**C reactive protein (mg/l)**	7.1 [3.0–16.7]
**Serum albumin (g/l)**	36.9±4.6
**Total cholesterol (mmol/l)**	5.02±1.28
**Triglycerides (mmol/l)**	1.72 [1.21–2.65]
**LDL cholesterol (mmol/l)**	2.96±1.00

*Note*: Qualitative variables expressed as number (percent). Quantitative variables expressed as mean ± SD for normally distributed variables or median [interquartile range] for non normally distributed variables;

*Abbreviations*: HDF, hemodiafiltration; HD, hemodialysis; min, minutes.

The values for CKM-derived CIs in the database (16,547 values) from 549 HD patients were used to develop the equation predicting CI using patient demographic parameters and dialysis dose. Age, gender, pre-dialysis serum creatinine concentrations (Cr_pre_) and _sp_Kt/V urea were selected as significant variables in the final model by using stepwise procedures. The equation to predict CI was determined as follows: 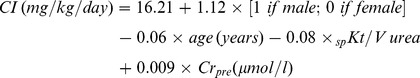
(Note. To evaluate CI using plasma creatinine concentration in mg/dl, divide the creatinine concentration term by 88.4).

**Figure 1:**
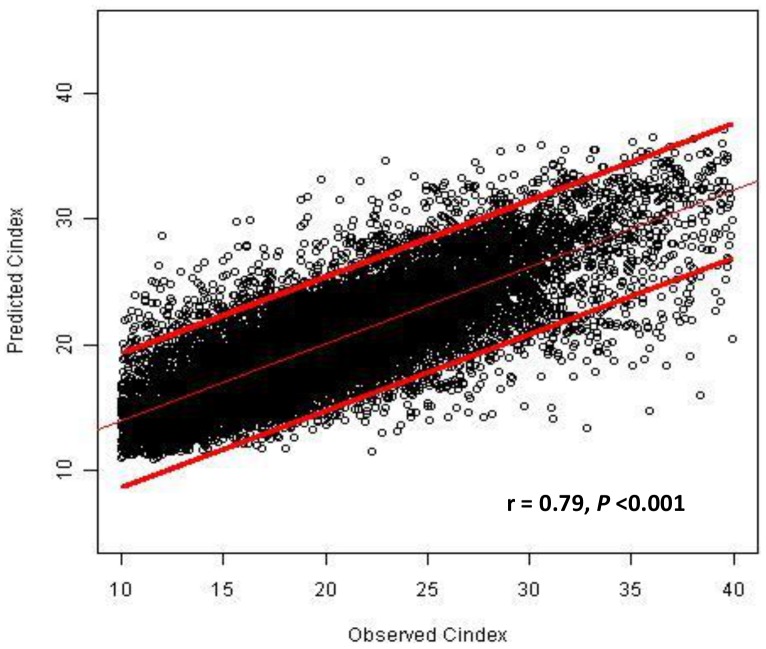
Scatterplot between observed creatinine index and predicted creatinine index. A scatterplot with a regression line and a 95% confidence interval for the line representing the relationship between creatinine kinetic modeling (CKM)-derived creatinine index (Observed Cindex) and creatinine index derived from the equation (Predicted Cindex).

**Figure 2:**
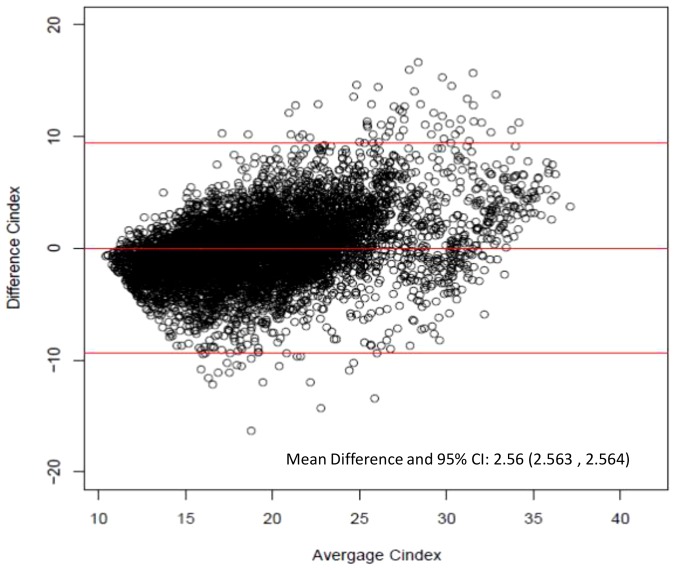
Bland-Altman plot of the difference between equation-derived versus creatinine kinetic modeling-derived creatinine index. The horizontal lines are the mean difference and 95% limits of agreement.

To estimate the validity of the equation, the measured CI using CKM was compared with the equation-derived CI in a large cohort of 549 HD patients. Equation-estimated CIs were closely correlated with CKM-derived CIs as illustrated in **Figure 1** (correlation coefficient  = 0.79, P-value <0.001). Bland-Altman analysis confirmed the excellent prediction of CKM-derived CIs using the equation (**Figure 2**). The mean prediction error was 13.47% (95% confidence interval  = 13.46–13.67) which is quite acceptable as a clinical tool. In addition, the developed equation precisely estimated CI in both men and women in all age groups (**Table 2**).

**Table 2:** Difference plot analysis comparing equation-estimated CI and CKM- derived CI according to gender and age groups.

	Limit of absolute error	Mean difference (95%CI)	Correlation (r)	pP-value
**All**	0 to 22	2.56 (2.56, 2.56)	0.79	<0.001
**Men**	0 to 19	2.62 (2.61, 2.62)	0.79	<0.001
**Women**	0 to 22	2.47 (2.47, 2.47)	0.76	<0.001
**Age <65 yrs.**	0 to 22	2.56 (2.56, 2.56)	0.79	<0.001
**Age ≥65 yrs.**	0 to 20	2.25 (2.25, 2.25)	0.75	<0.001

*Abbreviations*: CI, creatinine index; CKM, creatinine kinetic modeling; 95% CI, 95% confidence interval.

**Figure 3:**
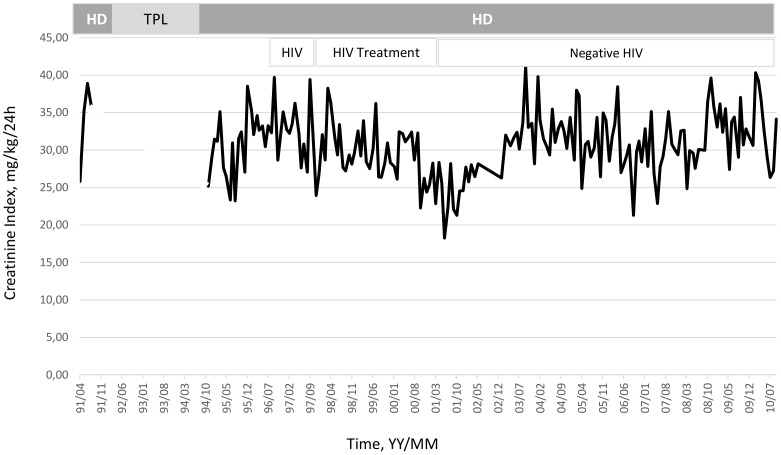
Illustration of clinical relevance and potential usefulness of the simplified CI formula. Case 1: Deteriorating effect of HIV treatment on CI in HD patient with recovery after stopping treatment (Pat. RB, Male, 32 yo, 68.8 Kg, _sp_KT/V 1.61±0.14, CI 30.5±4.7 mg/kg/24 h).

To illustrate the clinical relevance and potential usefulness of this simplified CI formula, a selection of few typical HD patients is presented in **Figures 3** **,** **4** **,** **5** **,** **6** **,** **7** **.** These cases were selected because they benefit from several years of follow-up and provide appropriate trend analysis. In summary, these cases illustrate different conditions: stable HD patient maintaining CI in normal range according to gender, age and physical activity; improvement in incident HD patient marked by a regular CI increase; deteriorating HD patient with muscle wasting due to intercurrent disease or protein energy wasting condition leading to death. The selection of cases underlines the clinical usefulness and applicability of simplified CI for comforting or alarming physicians on HD patient status. As shown, simplified CI may be easily implemented and used as a nutritional and physical activity assessment tool in the daily life of HD patients helping nephrologist seeking for dialysis adequacy. Time behavior follow-up of simplified CI is a complementary useful and cost effective tool that is easily implemented in the management of HD patient as a combined index with dialysis dose delivery.

**Figure 4:**
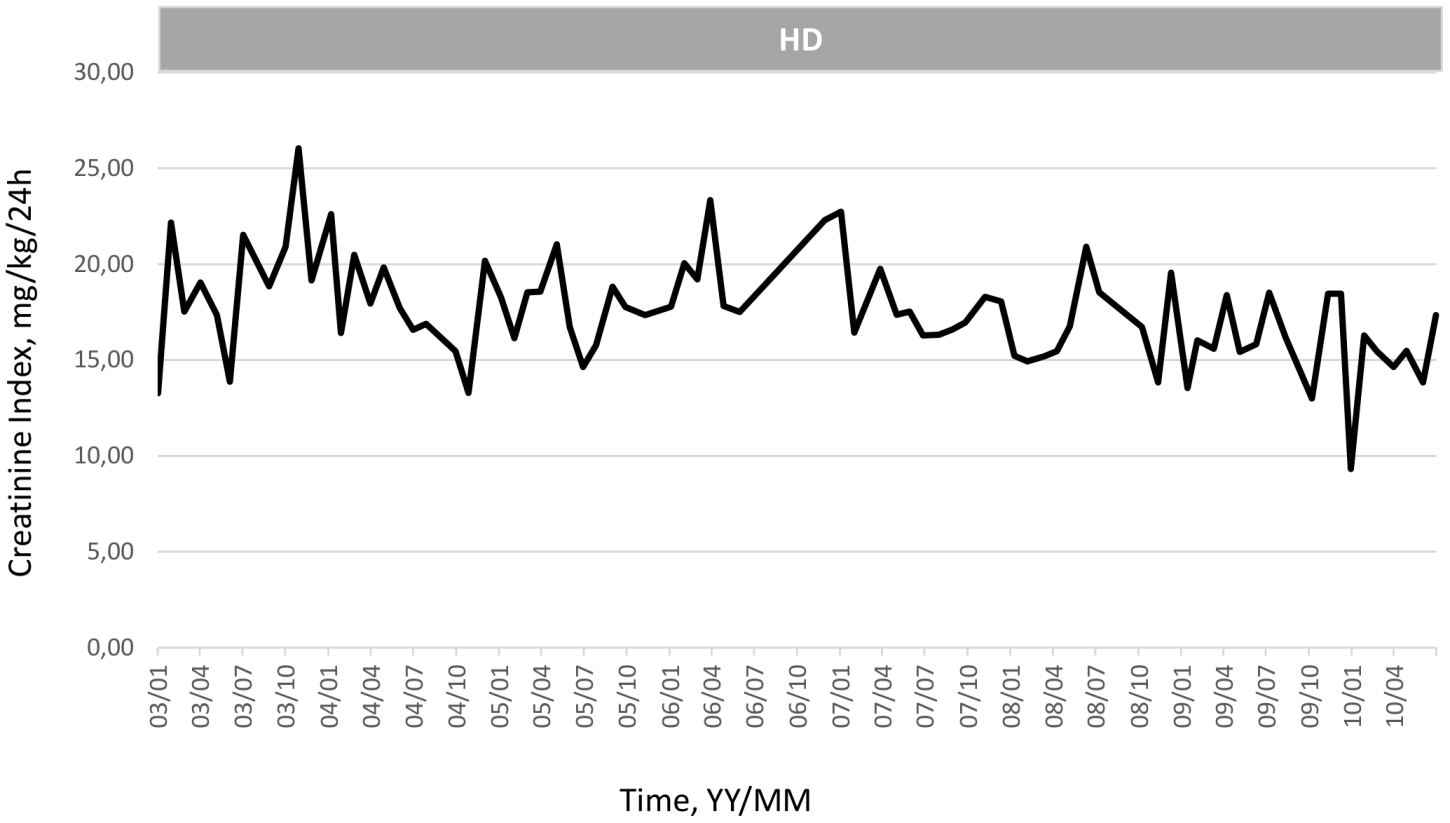
Illustration of clinical relevance and potential usefulness of the simplified CI formula. Case 2: Progressive decline of CI reflecting physiological muscle loss in elderly female HD patient (Pat. BM, Female, 70 yo, 61.7 Kg, _sp_KT/V 1.99±0.16, CI 17.6±2.7 mg/kg/24 h).

**Figure 5:**
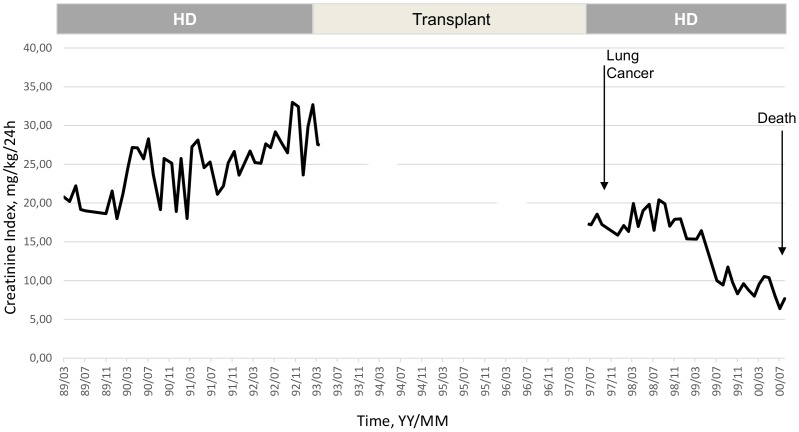
Illustration of clinical relevance and potential usefulness of the simplified CI formula. Case 3: Significant increase of CI and muscle mass after start of hemodialysis (1st phase), loss of muscle mass after renal transplant rejection and rapid deterioration of CI following lung cancer leading to death (Pat. BJ, Male, 58 yo, 76.5 Kg, _sp_KT/V 1.33±0.18, CI 20.2±6.7 mg/kg/24 h).

**Figure 6:**
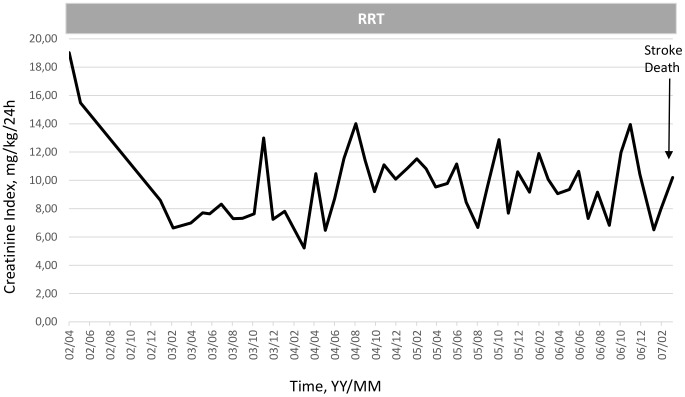
Illustration of clinical relevance and potential usefulness of the simplified CI formula. Case 4: Significant decline of CI and muscle mass in elderly woman after start of dialysis then stability maintenance of CI at low level until stroke and death (Pat. FM, Female, 88 yo, 72.2 Kg, _sp_KT/V 2.0±0.23, CI 10.2±3.5 mg/kg/24 h).

**Figure 7:**
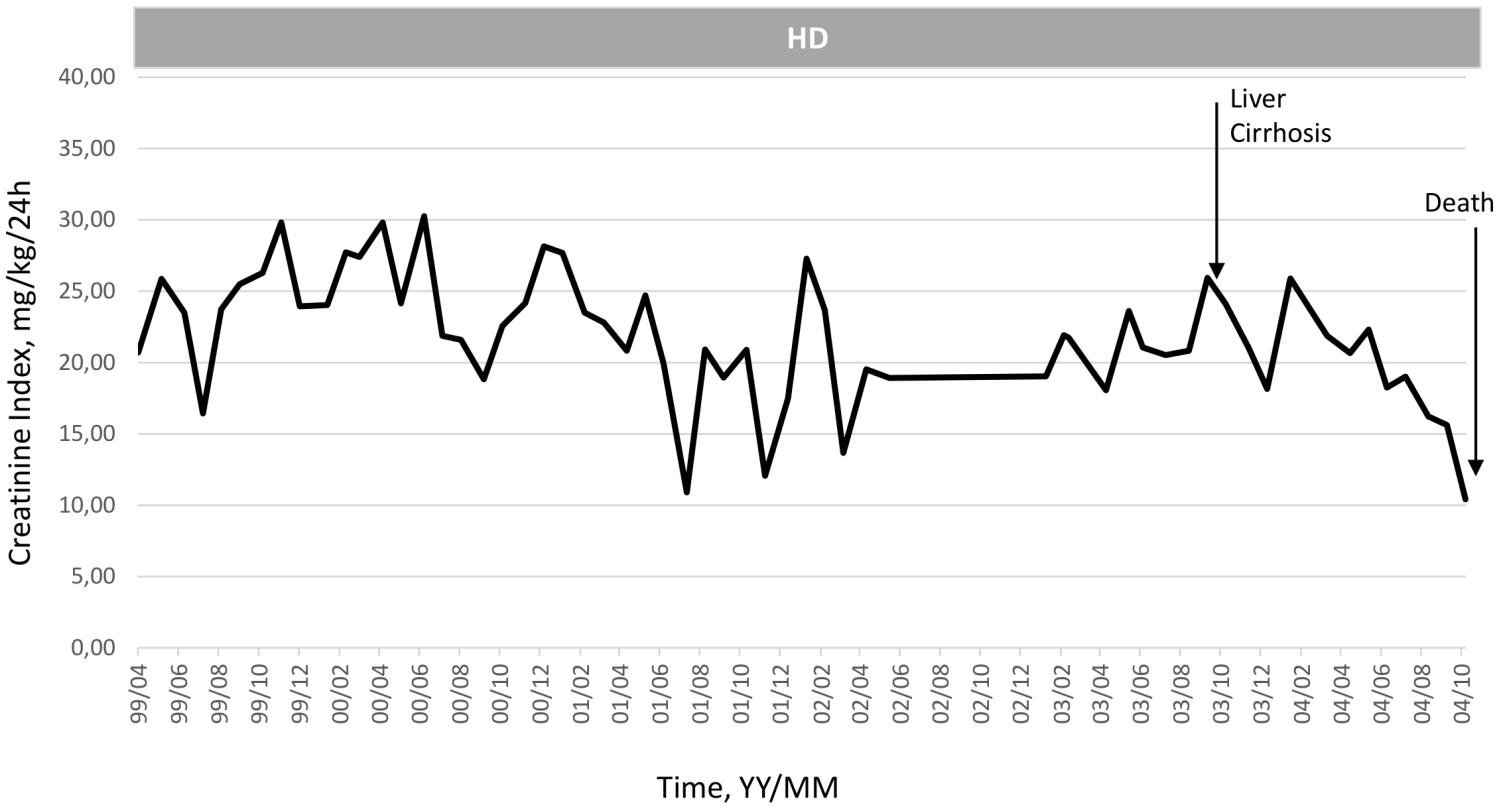
Illustration of clinical relevance and potential usefulness of the simplified CI formula. Case 5: Slow decline of CI reflecting muscle mass loss in young heart transplant man then followed by a rapid CI decline due to development of liver cirrhosis (peliosis hepatitis) leading to death (Pat. KJ, Male, 53 yo, 61.9 Kg, Heart transplant - CV Catheters, _sp_KT/V 1.62±0.41, CI 22.1±5.9 mg/kg/24 h).

## Discussion

In a large cohort of 549 hemodialysis patients followed more than 20 years with 16,547 assessments of CKM, we developed a highly simplified formula to predict CI only based on easily-attainable parameters of age, gender, pre-dialysis serum creatinine levels and _sp_Kt/V urea. The formula yielded an accurate estimation of CI with a strong correlation between CKM-derived CI and formula-estimated CI on the basis of both regression and Bland-Altman analyses. Moreover, the CI formula appeared accurate for predicting CI in both men and women in different age groups. Although the study population used for formula development includes mainly Caucasians, the formula is applicable to all HD populations. Ethnicity does not affect the validity of the formula because CI derived from CKM is based on creatinine mass balance determined from the sum of creatinine removal rate and metabolic degradation rate in the steady state that determines creatinine generation rate state [18]. In addition, one must recognize that the range of CIs values found in our population is sufficiently large to cover the wide spectrum of CIs values in different ethnicities.

Evaluation of muscle mass is an important measure for assessing protein nutritional status in HD patients [24]–[27]. Accurate methods for measuring LBM such as CT or MRI of muscle mass are expensive and their availability is limited in routine clinical practice. In addition, non-invasive techniques such as dual energy X-ray absorptiometry (DEXA) and bioelectrical impedance analysis (BIA) present some limitations in terms of muscle mass measurement. Both DEXA and BIA might not detect malnutrition in overhydrated dialysis patients due to overestimation of LBM [19], [28], [29]. Conversely, predialysis serum creatinine concentrations or CI obtained by CKM reflecting muscle mass have been recognized as significant predictors of protein nutritional status, all-cause and cardiovascular mortalities in HD patients [18], [20], [21], [30]–[33]. However, extended use of CI, a more sensitive indicator, based on CKM has been limited by two factors: firstly, the complexity of the mathematical modeling of formal creatinine kinetics for calculating CI is not easily applicable in daily practice; secondly, post-dialysis serum creatinine measurement is not performed in routine clinical dialysis practice for economical reasons. The simplified CI equation using age, gender, pre-dialysis serum creatinine concentrations and _sp_Kt/V urea was therefore developed to overcome these limitations and to provide an additional cost-effective tool for regular LBM nutritional assessment of HD patients. The equation yields serial estimates of CI and trend analysis of CI changes over time provides much more information on patient muscle mass and protein nutritional status than the absolute CI values. Significantly, muscle protein turnover rates are highly variable depending on age, gender and factors affecting protein synthesis and degradation [34]. CI declines with advancing age and is lower for women than men [18]. In addition, several conditions causing loss of muscle mass in chronic kidney disease patients such as catabolic illnesses [35], acidemia [36], [37] and resistance to anabolic factors (e.g. insulin, growth hormone and insulin-like growth factor-1) [38]–[40] could be easily and precisely detected by time reduction of CI values.

Five-year cohort studies of HD patients reported the prognostic significance of CKM-derived CI as an indicator of all-cause and cardiovascular mortalities in HD patients [20], [21]. Reduced CI (<22 mg/kg/day) was significantly associated with poor patient survival [20]. It could be argued from Bland-Altman analysis (**Figure 2**) that the accuracy of this new equation decreases above 30 mg/kg/day. However, the very small number of observations above this value (only 6%) may contribute to decrease estimator accuracy. And, as previously mentioned, only low CI or CI-derived LBM constitutes a high predictive value for mortality in HD population [20], [21] or CKD patients [25].

Clinically-relevant and interesting information provided by this simplified CI has been illustrated in selected cases from our cohort. As shown, simplified CI is useful to confirm stability of HD patients maintaining CI in a normal range according to gender and age, while a CI increase may illustrate nutritional improvement in HD patients after start of treatment or, more interestingly, a rapid CI decline would help clinicians to identify and intervene on HD patients undergoing wasting muscle mass.

The main limitation of this study lies in the absence of validation groups for this new equation. The predictive value of this simplified CI equation for long-term mortality in HD patients using routinely collected data (age, gender, pre-dialysis serum creatinine levels and _sp_Kt/V urea) needs to be assessed within large databases. The usefulness of this simplified formula within large databases is needed to ensure the reliability and accuracy of the formula for predicting long-term outcomes of HD patients. Two pilot studies have been conducted in two large cohorts of HD patients with promising results (DOPPS, MONDO) (unpublished data). As a perspective, the development of a website available for physicians may help to easily evaluate CI.

In summary, the simplified CI formula based on age, gender, pre-dialysis serum creatinine concentrations and _sp_Kt/V urea offers a simple, precise and cost-effective tool for CI estimation in HD patients. The predictive value of the equation-estimated CI on patient outcomes needs to be further validated in large epidemiological studies of HD patients but preliminary results appear quite promising.
